# *Escherichia coli* O157 infection on Scottish cattle farms: dynamics and control

**DOI:** 10.1098/rsif.2010.0470

**Published:** 2010-11-17

**Authors:** Xu-Sheng Zhang, Mark E. J. Woolhouse

**Affiliations:** Centre for Infectious Diseases, University of Edinburgh, Kings Buildings, West Mains Road, Edinburgh EH9 3JT, UK

**Keywords:** disease control, *E. coli* O157, Markov chain Monte Carlo, transmission dynamics

## Abstract

In this study, we parametrize a stochastic individual-based model of the transmission dynamics of *Escherichia coli* O157 infection among Scottish cattle farms and use the model to predict the impacts of both targeted and non-targeted interventions. We first generate distributions of model parameter estimates using Markov chain Monte Carlo methods. Despite considerable uncertainty in parameter values, each set of parameter values within the 95th percentile range implies a fairly similar impact of interventions. Interventions that reduce the transmission coefficient and/or increase the recovery rate of infected farms (e.g. via vaccination and biosecurity) are much more effective in reducing the level of infection than reducing cattle movement rates, which improves effectiveness only when the overall control effort is small. Targeted interventions based on farm-level risk factors are more efficient than non-targeted interventions. Herd size is a major determinant of risk of infection, and our simulations confirmed that targeting interventions at farms with the largest herds is almost as effective as targeting based on overall risk. However, because of the striking characteristic that the infection force depends weakly on the number of infected farms, no interventions that are less than 100 per cent effective can eradicate *E. coli* O157 infection from Scottish cattle farms, implying that eliminating the disease is impractical.

## 1. Introduction

*Escherichia coli* O157 is an important zoonosis that can cause severe illness in humans. Sporadic outbreaks of *E. coli* O157 infection occur worldwide. In Scotland, about 200 cases of infection in humans are reported annually, at the highest annual incidence rate globally during the past 20 years [1,2]. Cattle are the main reservoir host [3] and play a significant role in the epidemics of human infection [4]. Thus, understanding how *E. coli* O157 is spread among cattle farms and how it persists in the cattle population is crucial to controlling the infection in humans. Infection of cattle farms with *E. coli* O157 is typically transient, infection is usually harmless to cattle and infected cattle can become susceptible again. *E. coli* O157 is transmitted via the faecal–oral route and the main routes of infection are thought to be contaminated feed, water and grazing. Contamination of the farm environment relates in a complicated way to surrounding farms [5], with transport between farms thought to be by wild animals, birds and vehicles. To obtain the prevalence of *E. coli* O157 on Scottish cattle farms, two large surveys were conducted during the past decade: one from 1998 to 2000 (SEERAD; [6]), the other during 2002–2004 (IPRAVE; [7]). Both reported *ca* 20 per cent of cattle farms were affected, and together indicated that the level of infection has reached some approximate steady state [8] following its first appearance in the 1980s.

To understand the transmission dynamics of *E. coli* O157 infection on Scottish cattle farms, individual-based stochastic susceptible–infected–susceptible (SIS) metapopulation models were developed by Zhang *et al*. [9]. Different model variants that describe different mechanisms of spread of the bacterium among Scottish cattle farms were examined based on the IPRAVE survey data [7]. The IPRAVE survey was conducted between February 2002 and February 2004 and took faecal samples once from each of 481 cattle farms. Farms were visited in such a way that similar numbers were included from each of the six designated Animal Health Districts (AHDs) through Scotland and that AHDs were sampled evenly over time [8]. Although conducted over 3 years, the IPRAVE survey was actually cross sectional in nature. Data on the Council-Parish-Holding number were not recorded, so farms were matched by the *xy* coordinates to the DEFRA 2003 census data and DEFRA Cattle Tracing System (CTS) data. After matching, 461 of these farms were used for model fitting, among which 87 (18.9%) were infected (fig. 2 of Chase-Topping *et al*. [7]). It was found that the transmission dynamics of *E. coli* O157 infection on Scottish cattle farms can be most parsimoniously described by the model described below (§2) [9]. Because of the highly stochastic nature of the infection and recovery processes, there is a substantial amount of variation in estimated values of model parameters.

To explore this variation and its implications for the design of intervention strategies, in this study, we first employ a Markov chain Monte Carlo (MCMC) approach to generate their distributions. Based on these, we use simulations to examine the effectiveness of different modelled interventions. Measures such as vaccination [10], biosecurity or a combination of these [11,12] may be considered as options for controlling *E. coli* O157 infections in cattle. Several theoretical studies have investigated the expected impacts of such measures within a single herd [11–14]. These have provided useful information on how to deploy vaccination and improved biosecurity to reduce within-herd transmission, transmission from the wider environment and shedding rate and duration. In this study, we examine the impacts of intervention strategies at the level of the whole population of Scottish cattle farms, assuming that modelled levels of effectiveness of the interventions can be achieved in practice.

## 2. Methods

### 2.1. Model structure

The spread of *E. coli* O157 infection from one farm to another is due to movements of infected cattle among Scottish cattle farms and other routes such as environmental contamination or via other host species. Following Zhang *et al*. [9], we take the transmission rate owing to cattle movement to be proportional to the number of incoming animals that are infected. For other routes of transmission, the infectiousness of surroundings is nonlinearly dependent on the total number of infected farms, while the susceptibility of a farm is positively but nonlinearly related to the number of cattle present. Combining these, the overall probability at which farm *i* becomes infected on day *t* is given by2.1
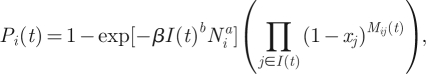
and the probability an infected farm recovers to become susceptible again per day is a constant:2.2
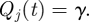


Here, *N*_*i*_ is the number of cattle on farm *i*, with the dimensionless nonlinear coefficient *a*, and *I*(*t*) is the number of infected farms at day *t* with the dimensionless nonlinear coefficient *b* [15,16]. *M*_*ij*_(*t*) is the number of cattle moved from farm *j* to farm *i* on day *t*. The quantities *M*_*ij*_(*t*) and *N*_*i*_ were obtained from DEFRA CTS [17] and the June 2003 Agricultural census data of the Scottish Government [18], respectively. *x*_*j*_ is the fraction of cattle infected on farm *j* that was sampled at each time step from the on-farm prevalence distribution reported for the IPRAVE survey data [19]. After matching IPRAVE survey farms with the 2003 census data and CTS data, our system comprises 13 704 cattle farms [9]. The model requires four parameters to be estimated: the transmission coefficient *β*, the nonlinear coefficients *a* and *b* and the recovery rate *γ*. The time step used in all simulations is 1 day.

### 2.2. Distribution of model parameter values

To estimate the values of model parameters and the distribution of these estimates, model simulations were compared with IPRAVE data (as presence or absence of infection) for 461 IPRAVE farms by calculating the natural logarithm of the likelihood2.3
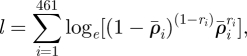
where *r*_*i*_ = 1 if farm *i* is recorded as positive, *r*_*i*_ = 0 otherwise. 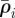 is the predicted probability that farm *i* is infected on a particular day. To get this probability, the system governed by equations (2.1) and (2.2) is employed. The system was assumed to be at steady state (consistent with epidemiological data from Pearce *et al*. [8]), which was obtained after ignoring the burn-in period of 9 years, then running for 3000 years, with the infection status of each IPRAVE farm recorded at the actually surveyed date once every 3 years. During the period of simulations, the 3 year movement data (from 2002 to 2004) were cycled. Then 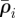 is approximated by the value of the total number of times that farm *i* is infected divided by 1000 (for details, see [9]).

The Metropolis–Hasting algorithm [20] was employed to generate the MCMC sampling of distribution of model parameters. The ‘proposal’ distributions for four parameters are independent. The candidate points for nonlinear coefficients *a* and *b* are independently sampled from normal distributions with standard deviation *σ*_a_ and *σ*_b_, respectively. So if the previous point for coefficient *a* is *a*_0_, the next point is sampled as *a* = *a*_0_+ *ξσ*_a_, where *ξ* is the standard normal variable, i.e. *ξ* ∼ *N*(0,1). The candidate points for transmission coefficient *β* and recovery rate *γ* are independently sampled from lognormal distributions with standard deviation *σ*_*β*_ and *σ*_*γ*__,_ respectively. For example, ln(*β*) = ln(*β*_0_) + *ξσ*_*β*_. For each set of parameter values (*β*, *γ*, *a*, *b*), the natural logarithm of the likelihood *l* is evaluated using equation (2.3). The new values of model parameters were accepted if2.4
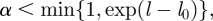
where *α* is a uniformly distributed random variable within the range [0,1], *l* and *l*_0_ are the natural logarithm of the likelihood for the new and old values of model parameters, respectively. The MCMC process governed by the Metropolis–Hasting algorithm (2.4) visits any possible set of model parameter values and reveals the uncertainty in parameter estimates. In view of its very low values, *β* was evaluated as log *β* throughout.

Four different Markov chains that started with different initial values of model parameters were run simultaneously and the Gelman–Rubin *R* statistic [21] was calculated to monitor the convergence of MCMC processes.

### 2.3. Effectiveness of control programmes

Based on the MCMC-generated distribution of model parameter values, the effectiveness of different control programmes was investigated using Monte Carlo simulations. We simply used the original distributions of parameter estimates generated by converged MCMC processes that reproduced the observed steady state. A total of 2486 sets of parameter values were randomly chosen from the total 56 094. For each set of sample values of model parameters, the model system starts with five randomly chosen infected farms and reaches some steady state after a burn-in period of 9 years [9,19]. Control programmes are introduced after the system has been at the steady state for 6 years, and the simulation is allowed to run a further 9 years to monitor the impact of interventions on the prevalence of infection. Several different strategies were examined: (i) no movement-related transmission between Scottish farms (achieved either through banning cattle movements or, more realistically, preventive measures targeted at cattle in transit between farms), (ii) reducing the transmission coefficient, *β*, (iii) reducing the duration that a farm is infectious to other farms, 1/*γ*, and (iv) targeting interventions (i)–(iii) not at the whole population of farms but at farms selected on the basis of herd size or other risk factors.

To compare the effectiveness of the non-targeted interventions (i)–(iii), we proceeded as follows. For reduction of the transmission coefficient *β* (e.g. improved biosecurity or vaccination), we consider various levels of ‘control effort’ (*c*), and the transmission coefficient is reduced to (1 − *c*)*β*. For a reduction in the period for which a farm is infectious (e.g. case finding and treatment, quarantine or culling), the recovery rate increases to *γ*/(1−*c*) for all farms. For reductions in movement-related transmission, we considered only an all-or-none effect of control. We also consider various combinations of these interventions.

For targeted interventions, control effort, *c*, is re-defined as the proportion of farms where the intervention is applied (with an assumed 100% effectiveness on the targeted farms). On these farms, as appropriate, we allow no movements on or off, set *β* = 0 and/or set 1/*γ* = 0. Four methods of selecting farms were considered: (i) based on farm size, *N*_*i*_, (ii) based on the estimated probability that a farm is infected, i.e. 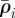 in equation (2.3), (iii) based on whether a farm was infected or not immediately before control was implemented, and (iv) random targeting.

## 3. Results

### 3.1. Estimates of model parameters

**Figure 1.**
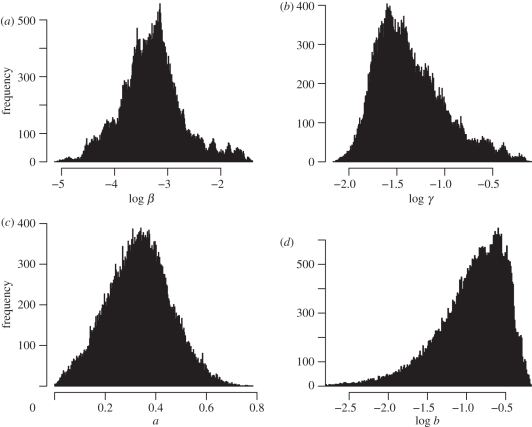
Univariate distributions of estimates of model parameters: (*a*) log *β*, (*b*) log *γ*, (*c*) *a*, and (*d*) log *b*. For better illustration, especially for small values, both *γ* and *b* are transformed as log *γ* and log *b*. The data are from four MCMC that started from different initial values of model parameters. After removing the first 5000 iterations for each chain, the iterations for four MCMC processes totalled 56 094.

After removing the first 5000 iterations (assumed as the burn-in period), we obtained four convergent MCMCs of a total 56 094 iterations, which were characterized by the values of Gelman–Rubin *R* statistics [21]: 1.02, 1.03, 1.02, 1.00 for the four model parameters log *β*, *γ*, *a*, *b* and 1.02 for the natural log of the likelihood (*l*). The MCMC samplings of four univariate frequency distributions for model parameters are shown in figure 1. The distributions of both coefficient *a* and log *β* are very close to normal, while distributions of logarithm of both recovery rate *γ* and coefficient *b* can be approximated by gamma distributions with the shape parameter larger than unity. From the univariate distributions, we obtained the estimates of modal values and 95% percentiles, which are given in table 1.

**Table 1.** Summary of the four univariate distributions and the joint distribution of four parameters of the model described by equations (2.1) and (2.2). To obtain the four-dimensional distribution of parameters log *β*, log *γ*, *a* and log *b*, the whole space occupied by 56 094 points has been divided into 20^4^ blocks. These are arranged in the descending order of frequencies. The values of the highest block count give modes, and the lower and upper of the 95% CI are set to the lower and upper parameter values represented by the blocks that must be included to encompass 95% of the total count. The intervals used for log *β*, log *γ*, *a* and log *b* are 0.19, 0.10, 0.039 and 0.13, respectively. Central values for each block are referred to throughout.

	log *β*	*γ*	*a*	*b*
univariate distribution				
mode	−3.15	0.025	0.351	0.269
95% CI	(−4.43,−1.88)	(0.013, 0.317)	(0.086, 0.583)	(0.010, 0.472)
joint distribution				
mode	−3.54	0.032	0.372	0.181
95% CI	(−5.03,−1.49)	(0.008, 0.708)	(0.020, 0.763)	(0.002, 0.578)

To obtain the joint distribution of the four model parameters, the entire four-dimensional area was divided into 20 × 20 × 20 × 20 blocks (giving intervals of 0.19, 0.10, 0.039 and 0.13 for log *β*, log *γ*, *a* and log *b*, respectively). The smallest set of blocks containing 95 per cent of iterations was identified to determine the 95 percentiles for the joint distribution. The modes and 95 percentiles of the joint distribution are also listed in table 1. The modes of parameter values obtained from univariate and joint distribution showed good agreement, but the 95 percentiles tend to be wider for the latter, partly owing to the coarser scales used and partly owing to uncertainty regarding the precise location of the ‘edges’ of the joint distribution.

The rate of transmission is determined by the values of *β* and the nonlinear coefficient *b. b* is significantly less than one, but not significantly greater than zero (table 1). The transmission rate is also nonlinearly weighted by herd size through the nonlinear coefficient *a*. The 95% confidence interval (CI) for *a* does not include zero (table 1), demonstrating a significant effect of herd size on the probability of infection.

**Figure 2.**
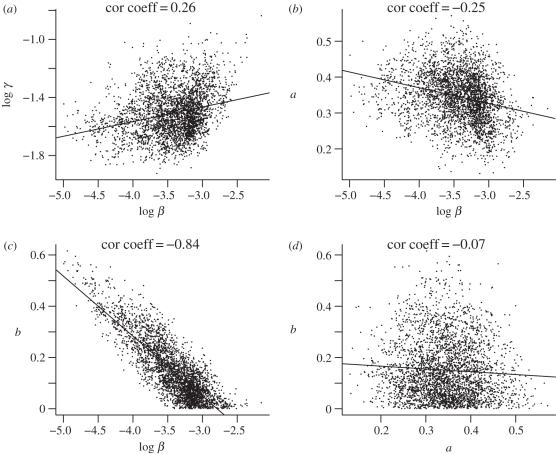
Correlation between model parameters generated by the MCMC method: (*a*) log *γ* and log *β*, (*b*) *a* and log *β*, (*c*) *b* and log *β*, and (*d*) *b* and *a*. The scattered points are the 3360 MCMC iterations that have negative natural logarithm of likelihood less than 215.8, the lines are the best-fit regressions and the numbers are the correlation coefficients. If all 56 094 MCMC iterations are included the correlations remain roughly the same.

**Figure 3.**
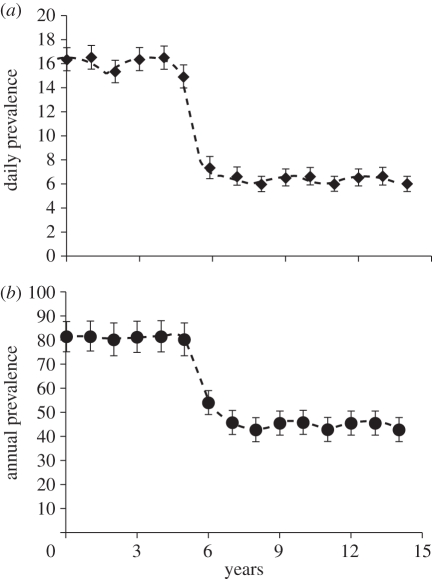
Dynamics of infection prevalence following the introduction of control measures. Two prevalence estimates are shown: (*a*) point prevalence (based on the average of monthly values); (*b*) annual prevalence (farms infected at any time during the year). The control measure reduces the transmission coefficient to *β* = 0 for farms of size equal and larger than 130 (i.e. control performed on the largest 39% farms). Error bars denote the standard deviation.

There are some correlations among different parameters (figure 2). For example, log *β* is positively correlated with the log recovery rate, log *γ* (figure 2*a*), and negatively correlated with the nonlinear coefficients *a* (figure 2*b*) and *b* (figure 2*c*). However, there is only a weak correlation between coefficients *a* and *b* (figure 2*d*). Therefore, different sets of parameter values can reproduce the same steady-state prevalence of about 19 per cent among the IPRAVE farms. This gives rise to a large variation in values of model parameters and therefore a fairly flat distribution of likelihood versus parameter values. At steady state with the baseline parameter values, the point prevalence for the entire system remains at about 16 per cent (which corresponds to 81% of farms being positive at some point during a given 1 year period, i.e. ‘annual prevalence’; figure 3). It is worth mentioning the difference between the prevalence on IPRAVE farms (19%) and that on the whole farms (16%) at steady state. Noting that larger herds are more likely to become infected, the difference results from the fact that the IPRAVE survey excluded smaller cattle farms [7].

### 3.2. Interventions

Based on the model (equations (2.1) and (2.2)) and the MCMC-generated distribution of model parameters, the Monte Carlo simulations were used to model changes in prevalence given different interventions. For illustration, figure 3 shows the influence on the prevalence achieved by setting the transmission coefficient *β* = 0 on the farms of herd size ≥130 (about 39% of the largest farms). With execution of the intervention, the average daily prevalence decreases from 16 per cent to about 6 per cent and annual prevalence decreases from 81 to 44 per cent. In the following, we report only infection prevalence at the new steady state under control, which is typically reached within 2 years.

**Figure 4.**
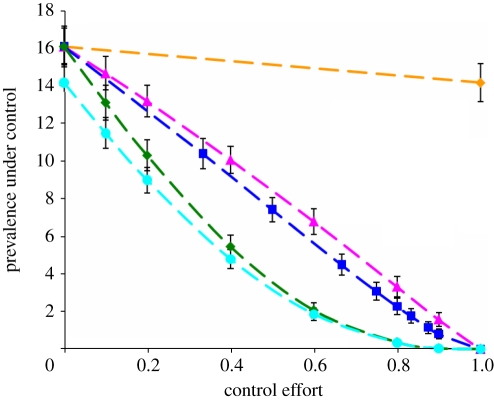
Effectiveness of non-targeted interventions. Five different interventions are displayed: no movement-related transmission, reducing transmission coefficient, reducing infectious period (i.e. increasing recovery rate), reducing transmission coefficient and infectious period simultaneously and combining all three. The prevalence was obtained after interventions have been performed for 9 years (figure 3). The mean values were obtained by averaging over the last 3 years across 2486 sets of original model parameter values from the converged MCMC process. Error bars denote standard deviations. Dashed orange line, no movements; dashed pink line, reduce *β*; dashed dark blue line, increase *γ*; dashed green line, joint control with movements; dashed light blue line, joint control without movement.

The simulation results for the various non-targeted interventions are shown in figure 4. It is clearly shown that reducing cattle movement between farms is least effective and shortening the infectious period (i.e. enlarging the recovery rate) is most effective in controlling *E. coli* O157 infection on Scottish cattle farms. The difference between shortening the infectious period and reducing the transmission coefficient is not substantial: for example, with the same control effort *c* =50 per cent, the prevalence decreases from 16 per cent to about 7 and 8 per cent respectively. Interventions that reduce both the transmission coefficient and the recovery rate result in a greater reduction in the prevalence of *E. coli* O157 infection. Additionally eliminating movement-related transmission significantly increases the effectiveness of the control only when the control effort is small.

**Figure 5.**
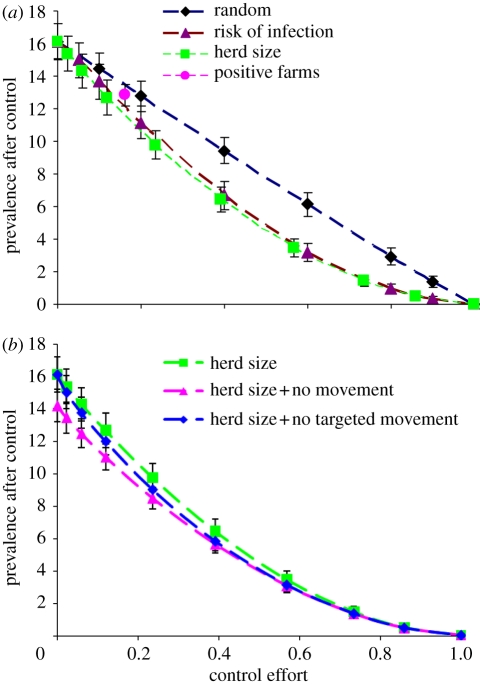
Effectiveness of targeted interventions where the selected farms assume a transmission coefficient *β* = 0. (*a*) Targeting farms based on herd size, estimated risk of infection and known presence of infection (positive farms). For comparison, the targeted control based on randomly chosen farms is also shown. The random targeted intervention is very similar to the non-targeted intervention that reduces the transmission coefficient by the control effort (cf. figure 4). (*b*) Effect of combining targeted interventions (based on herd size) with no movement-related transmission for either the targeted farms or all farms. Error bars denote the standard deviation.

The effectiveness of different targeted interventions is shown in figure 5. We focus on the impact of reducing transmission rates, *β*, with or without eliminating movement-related transmission. Setting the infectious period (1/*γ*) = 0 days on targeted farms has similar effects to setting *β* = 0. Random targeting has similar impacts to partial, non-targeted interventions (cf. figure 4). Interventions targeted at larger herds are considerably more efficient (figure 5*a*), reflecting that the susceptibility of a farm is a function of herd size (equation (2.3)). Eliminating movement-related transmission can increase effectiveness if only a minority of farms are targeted (figure 5*b*). Eliminating movement-related transmission to and from the same targeted farms is almost as effective unless the control effort is small (figure 5*b*). If interventions are targeted only at farms known to be infected (roughly 16% of all farms), the prevalence of infection only decreases to about 13 per cent. Interventions targeted at farms based on the estimated probability of being infected, 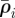, are only slightly less effective than those targeted on the basis of herd size (figure 5*a*). This is because, in practice, herd size accounts for most of the variation in risk. In all cases, however, to eliminate the infection from Scottish cattle farms, 100 per cent control effort is needed (figures 4 and 5).

## 4. Discussion

In this study, we obtained the MCMC-generated distribution of model parameters for transmission dynamics of *E. coli* O157 on Scottish cattle farms and further examined the effectiveness of various control strategies in reducing *E. coli* O157 infection. We found that, despite the considerable uncertainty in individual parameter estimates, there was much less uncertainty in the expected impacts of interventions, hence the small standard deviations in figures 4 and 5. This reflects that the different parameter combinations are constrained to reproduce the same observed prevalence. This means that it is possible to make much more robust predictions about which interventions are most effective than might have been anticipated. We have shown that interventions targeted at high-risk farms are more effective than non-targeted interventions. However, interventions that are less than 100 per cent effective cannot eradicate the infection; this reflects the inclusion of the nonlinear coefficient *b* in the model, which means that the force of infection is less than proportional to the total number of positive farms in the population (see below).

We found that while parameter estimates have wide credible intervals, the modes of both univariate and joint distributions are comparable with the maximum-likelihood estimates [9]. The difference might reflect the highly stochastic nature of the dynamical process and the uncertainty in the model fittings (fig. 3 of [9]). In contrast to the maximum-likelihood method, the MCMC method can easily generate credible intervals for parameter estimates (figures 1 and 2).

The susceptibility of a farm is related to its herd size with the nonlinear coefficient having a mode of 0.35 (0.37) as estimated from univariate (joint) parameter distribution, which is significantly less than unity but significantly greater than zero. This indicates that larger herds have higher probability of becoming infected [9]. The mode of the recovery rate is about 0.025 (0.032) per day (therefore the average infectious period is about 40 (31) days), which is consistent with data from smaller scale longitudinal studies [22]. The transmission rate nonlinearly depends on the number of infected farms with a coefficient of a mode 0.27 (0.18), which is also significantly less than unity. This implies that the simple assumption that farms are ‘well mixed’ is not adequate [9]. Indeed, from these results, *b* is not significantly greater than zero; *b* = 0 would imply that other infected farms do *not* pose a direct risk to a susceptible farm and that the main source of infection was some other reservoir of infection.

Although cattle movements do play a role in the epidemiology of other infectious diseases of livestock [23,24], reducing, or even eliminating, movement-related transmission has, at best, a modest impact on the steady-state prevalence of *E. coli* O157. This implies that cattle movements alone are insufficient to maintain infection in this population [9,19]. Reducing movement-related transmission can, however, play a modest role in supplementing other interventions (figures 4 and 5). Our simulation study also shows that the seasonality seen in prevalence before intervention is consistent with the patterns in cattle movement among farms [9,19], because it dies away when there is no movement-related transmission (data not shown).

As *E. coli* O157 is deposited in and can even reproduce in places such as water troughs, food stores, slurry, faeces and pen floors [13], the farm environment acts as a source of *E. coli* O157 infection both for cattle on the farm and for other farms. This contamination allows the spread of infection to other farms (by any route other than the movement of infected cattle), which is represented by the transmission coefficient *β*. Reducing the between-farm transmission rate *β* could be achieved by measures such as improved biosecurity or, in principle, by vaccination [10]. However, unless *β* is reduced to zero, infection is not eradicated. This is in contrast to the behaviour of simple SIS models [25,26] and arises because the coefficient *b* is less than one. The prevalence of infection can also be reduced by decreasing the infectious period (i.e. increasing the rate of recovery *γ*). Increasing *γ* could be achieved by reactive control measures implemented on affected farms, e.g. removal or isolation of infected cattle, changes in feed or management practices [27–29]. Again, however, it may not be practical to eradicate infection through such measures.

If one or more interventions can reduce *β* and increase *γ* together, then this is very effective in bringing the prevalence to a lower level; its effectiveness can be further increased if movement-related transmission is also eliminated (figure 4).

Interventions targeted at high-risk farms are, in general, more efficient than non-targeted interventions (figures 4 and 5). However, the improvement in efficiency is relatively modest, reflecting that the risk of infection varies only moderately across the population of farms [9]. Targeting based on herd size is almost as efficient as targeting based on the estimated probability of infection, reflecting that size is the most important risk factor so far identified. Interestingly, targeting farms based on the presence of infection is somewhat less efficient (figure 5*a*), reflecting the stochastic nature of the infection process, which makes the presence of infection at a given time point an unreliable guide to the underlying risk.

This analysis suggests that eradication of *E. coli* O157 in Scottish cattle farms through control measures directed at cattle is not practicable. However, a sustained control effort, especially if targeted at the highest risk farms, could, in principle, lead to a reduction in the prevalence of infected farms and so to a reduction in the risk of human exposure to infection.
